# A Case of Common Variable Immunodeficiency with CREBP Gene Mutation without Rubinstein Taybi Syndrome Features

**DOI:** 10.1155/2022/4970973

**Published:** 2022-07-04

**Authors:** Ugur Musabak, Serdar Ceylaner, Tuba Erdogan, Ebru Sebnem Ayva

**Affiliations:** ^1^Division of Immunology and Allergy, Baskent University, Faculty of Medicine, Ankara, Turkey; ^2^Department of Medical Genetics, Lokman Hekim University, Ankara, Turkey; ^3^Department of Pathology, Baskent University, Faculty of Medicine, Ankara, Turkey

## Abstract

Hypogammaglobulinemias, based on inborn errors of immunity, are primary immunodeficiencies (PIDs) that can also be diagnosed for the first time in adulthood. Common variable immunodeficiency (CVID) is a multifactorial disease often symptomatic due to antibody deficiency. In addition, some PIDs are classified into the category of immunodeficiencies with syndromic features due to their accompanying clinical findings unrelated to immunity. In this article, a patient with CVID who was diagnosed in adulthood and who was revealed to have a mutation specific to Rubinstein–Taybi syndrome and clinical features reminiscent of this syndrome only after molecular tests was presented.

## 1. Introduction

Primary immunodeficiencies (PIDs), a group of rare diseases, are caused by defects or dysfunction of the human immune system [1]. The characteristic manifestations of PIDs are chronic, serious, or life-threatening infections. These disorders, also called as inborn errors of immunity (IEI), are mainly divided into 10 subgroups according to the affected component/components of immunity [2]. Predominantly antibody deficiencies (PADs) are the most prevalent PIDs among these subgroups. In addition, the patients with PIDs who have some congenital anomalies are classified into a subgroup of PIDs with syndromic features. The frequency of PIDs in this subgroup is less than those of PADs. Common variable immunodeficiency (CVID) is one of the PADs in which a heterogeneous clinical manifestation is presented. Herein, we report a rare case with CVID who had some clinical features of Rubinstein–Taybi syndrome (RTS).

## 2. Case Presentation

**Figure 1:**
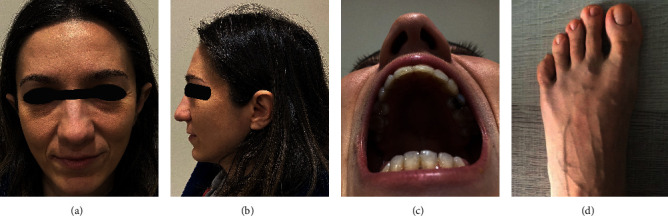
The patient with high arched palate, missing teeth, and wide big toes.

A 38-year-old female patient was admitted to our immunology outpatient clinic due to nodular lymphoid aggregates detected in the upper gastrointestinal tract examination performed for chronic diarrhea. In the patient's history, it was learned that after the age of fifteen, she had five or six sinusitis attacks per year and had pneumonia twice in total. In addition, she also had frequent and prolonged watery diarrhea in the last two years and was treated for long-term vaginitis. The patient was born at term by normal delivery. There was no mental or developmental retardation in her childhood compared to her peers. The patient's school success was consistently high. The patient, who is a pharmacist, has a 10-year-old boy. The patient's parents are not relatives. The patient, the only child in the family, did not have a history of recurrent infections or a dysmorphic disease in her parents or close relatives. On physical examination, the patient's height was 160 cm, her weight was 46 kg, and the body mass index was 17.9. On physical examination, she did not have the clinical features of Rubinstein–Taybi syndrome-like mental retardation, postnatal growth deficiency, microcephaly, and dysmorphic facial features. But she had a high arched palate and slightly wide big toes [Figure 1]. Twenty years ago, the patient underwent orthodontic treatment due to crooked and misplaced teeth. Therefore, the tooth arrangement was seen as normal. However, her teeth were missing due to tooth extraction during dental treatment. Other systemic examination findings were normal. All routine biochemical parameters measured in venous blood were within normal limits except for the total protein level [5.1 g/L (6.2–8.3)]. A complete blood count (CBC) with differential was respectively found as white blood cell: 10.8·103/mm^3^ (4.5–11.103), neutrophil: 4.6·103/mm^3^ (2–7.8·103), lymphocyte: 2.7·103/mm^3^ (1–4·103), monocyte: 0.5·103/mm^3^ (0–1·103), eosinophil: 2.7·103/mm^3^ (0–1·103), basophil: 0.1·103/mm^3^ (0–0.2·103), haemoglobin: 14.7 gr/dL (12.5–16), haematocrit: 43.2% (37–47), and platelet count: 156·103/mm^3^ (150–400·10^3^). Acute phase reactants were slightly increased than their reference values, such as C-reactive protein (CRP): 12 mg/L (0–5) and erythrocyte sedimentation rate (ESR) 22 mm/hour. Serum tissue transglutaminase antibody levels in IgA (tTg-IgA) and IgG (tTg-IgG) isotypes were lower than 2 RU/mL (<20: negative). Although antimicrosomal antibodies and antithyroglobulin antibodies were found to be increased as, respectively, 52.6 IU/mL (<5, 6) and 94.1 IU/mL (<4), the patient's thyroid-stimulating hormone (TSH) was at a normal level of 1.4 mU/L (0.3–4.9).

Serum levels of all major immunoglobulin isotypes were low at the time of diagnosis. Respectively, IgG: 0.27 g/L (6.5–16.3), IgA: 0.02 g/L (0.6–4.2), IgM: 0.12 g/L (0.3–2.9), and total IgE: 11 IU/mL (<87). While the patient's blood group was A Rh +, the anti-B antibody titer was lower than 1:8. The anti-HB antibody level of the patient, who was vaccinated 5 years ago with hepatitis B, was 750 U/L (<10). The percentages of lymphocyte subsets were found within reference ranges as CD3: 74.8%, CD4: 28.1%, CD8: 33.4%, CD19: 17.9%, CD45: 100%, CD3-CD16+CD56+: 6,8%, respectively.

Sixty-seven leukocytes (0–5) were detected in each field in the complete urinalysis. The stool of the patient was watery in appearance. However, no parasites, parasite eggs, or bleeding were observed in the microscopic examination of the stool. The patient's fecal calprotectin level was >1800 mcg/g of stool (>50).

No splenomegaly or LAP was detected in the whole abdominal ultrasonography, except for the slightly enlarged liver. In addition, no abnormal findings were seen in direct anterior-posterior and lateral chest radiographs.

**Figure 2:**
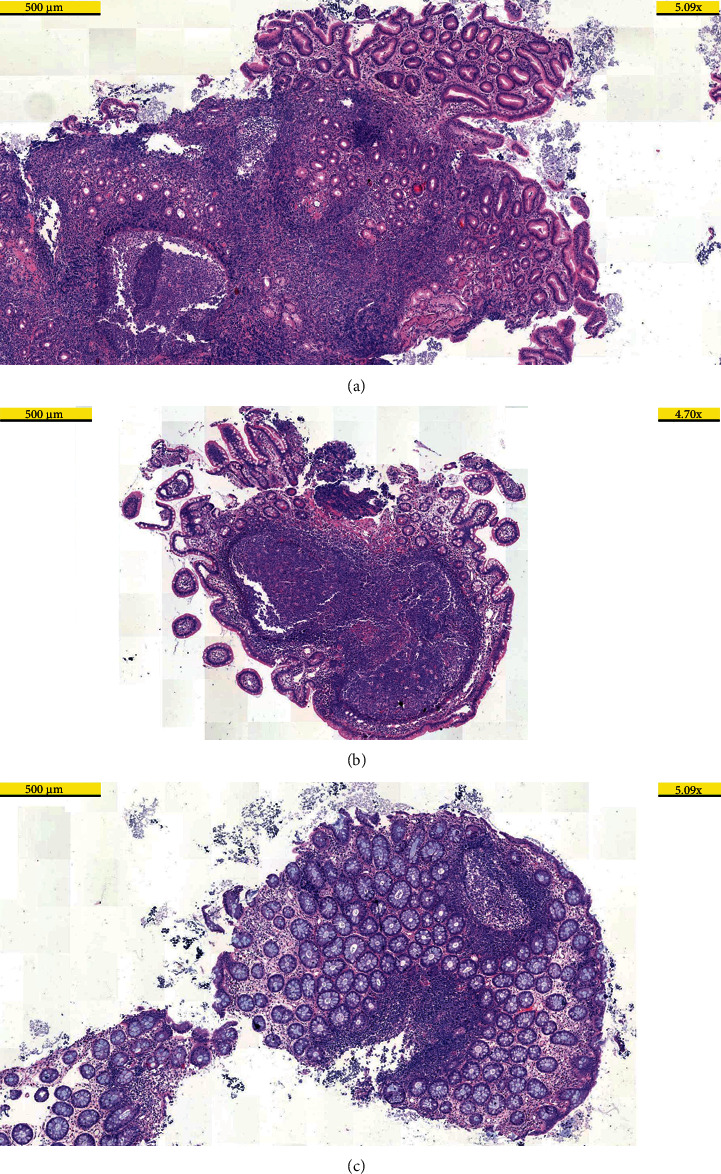
Hyperplastic lymphoid follicles with prominent germinal centers, in keeping with nodular lymphoid hyperplasia. Stomach (a), small intestine (b), and colon (c) biopsies with haematoxylin-eosin staining.

In the endoscopic examination of the upper gastrointestinal tract, the findings were consistent with pangastritis and nodular bulbitis. The rectum, cecum, and ascending colon mucosa were observed as mottled, hyperemic, and partially pale in the colonoscopic examination. Multiple millimeter-sized nodules along 20–25 cm were detected in the terminal ileum. In the histopathological examination of the biopsies, while chronic erosive gastritis accompanied by intestinal metaplasia was observed in the stomach, *Helicobacter pylori* was observed in abundance. Multiple nodular lymphoid hyperplasia (NLH) with prominent germinal centers were detected in the mucosa of the stomach, duodenum, terminal ileum, and colon, while an inflammatory reaction characterized by an increase in eosinophils was observed in the mucosa of the duodenum, terminal ileum, and colon (>50, >50, >100 per HPF (high-power field), respectively) (Figure 2).

When the previous biochemical tests of the patient were examined retrospectively, it was determined that the total protein levels were consistently low. As the patient had profound hypogammaglobulinemia, serum immunoglobulins were measured to confirm once again before treatment. The patient's previous low protein levels were interpreted as the result of low immunoglobulin levels. The patient was diagnosed as CVID in accordance with the European Society for Immunodeficiencies (ESID) criteria [3].

Whole exome sequencing (WES) was performed using next-generation sequencing (NGS) technology for the molecular diagnosis of the patient. A heterozygous pathogenic variant was detected in exon 31 of the CREBBP gene [NM_004380.3; c.5552G > A (p. Argl851His)], which is specific for Rubinstein–Taybi 1. In addition, HLA alleles have been identified in the patient, which increase the likelihood of gluten enteropathy (HLA-DQ2 : DQA1 *∗* 05 : 05 : 01 and -DQB1 *∗* 02 : 02 : 01). Our patient is married and has a 10-year-old boy. Molecular tests were also applied to other family members so that we could guide their future plans. It was seen that her son also had the same pathogenic variant in one allele of the CREBBP gene. There is just one case with this in the Genome Aggregation Database (GnomAD) and Genomes database. The UniProt database showed that this variant was 63% pathogenic for the CBP_HUMAN region of interest. Pathogenicity prediction was conducted based on 9 different computational methods. A different variant in the same codon was classified as pathogenic in Clinvar Database. That's why, we classified this variant as a “likely pathogenic variant” due to ACMG criteria.

In direction with the recommendations of international drug agencies, immunoglobulin (IVIG) treatment was initiated at the dose of 600 mg/kg every three weeks due to hypogammaglobulinemia [4]. In addition, oral budesonide, a topically acting synthetic steroid, at three daily doses of 3 mg and oral methylprednisolone (MP) at a single daily dose of 0.5 mg/kg, were used for the treatment of NLH [5]. The dose titrations of these drugs were adjusted according to the patient's clinical response. Vitamin D and Calcium supplements were also given to prevent the adverse effect of glucocorticoid therapy on bone metabolism.

## 3. Discussion

CVID is a heterogeneous PID that develops autoimmunity, malignancy, and granuloma formation in the course of the disease, except for recurrent infections [5, 6]. Although recurrent infection is the hallmark of the CVID, noninfectious manifestations may appear before the infection. This disease, with an incidence of between 1/25,000 and 1/50,000, occurs after the age of 4 or in adulthood. Immune cytopenia is the most common autoimmune complication developing in CVID. The autoimmune diseases that mainly affect the joints, skin, and gastrointestinal tract can also develop in this disease (e.g., rheumatoid arthritis, systemic lupus erythematosus, psoriasis, vitiligo, and immune enteropathies). The most common malignancies developing based on CVID are gastric cancer and lymphomas.

Noninfectious inflammatory enteropathies like those of ulcerative colitis, Crohn's disease, and celiac disease occur in 10 to 12% of patients with CVID [6, 7]. In patients with gastrointestinal complications, the clinical course is relatively severe, and the prognosis is poor due to malabsorption and weight loss. Accordingly, in the previous report, the common gastrointestinal tract conditions were gastritis/duodenitis (73.1%), NLH (30.8%), and chronic diarrhea (19.2%) in our cohort with CVID [6]. In addition, chronic diarrhea and NLH were significantly more common in the patients with a low body weight than in those patients with an ideal body weight (83.3% vs. 0%, *p*= not applicable: 83.3% vs. 15%, *p*=0.001, respectively).

Herein, we present a patient with CVID who had noninfectious inflammatory complications throughout the gastrointestinal tract. The patient's endoscopic and histopathological findings supported the presence of an obvious inflammatory process in the mucosa. Alleviation of symptoms with a gluten-free diet showed that the patient had a disease similar to celiac disease. Compatible with clinicopathologic findings, HLA-DQ2 alleles showing the likelihood of gluten enteropathy were detected in the case.

Interestingly, instead of variants identified in immunodeficiencies, a heterozygous pathogenic variant in the CREBBP gene which is specific for RTS was detected in WES analyses performed to define the genetic aetiology. Because of this, the patient was reexamined by the medical geneticist with respect to showing the clinical features specific to RTS. As a result of a careful examination, mild dysmorphic features consistent with this syndrome were found in the patient. She did not have mental retardation, which is a characteristic feature of RTS. Even the case was a graduate of the faculty of pharmacy.

Although recurrent infections are seen in a significant portion of the patients, there has been no comprehensive study investigating the immune system in patients with RTS to date. In a recently published meta-analysis, previously published articles on RTS in the literature were reviewed by Saettini et al., and clinical and immunological data of a large cohort of 97 patients with RTS were analyzed in detail [8]. In result, one in five patients with RTS were found to have hypogammaglobulinemia having clinical manifestations consistent with CVID or ALPS. As in the patient we presented, it was shown that the diagnosis of hypogammaglobulinemia was delayed until adulthood in all 3 patients in this cohort.

It is well known that orofacial dysmorphism, growth retardation, and intellectual disability are characteristic clinical features in RTS. However, not all of these findings may typically be found in all cases, and each patient may present with a different clinical phenotype. In a cohort with RTS consisting of 11 individuals described by Menke et al., some patients had a few signs compatible with RTS, such as growth retardation and less characteristic low-set ears or micrognathia [9]. Nine patients had apparent intellectual disability (82%), while none of them had the classical facial RSTS features, the truly broad or angulated thumbs, and halluces, or the broad distal phalanges of the fingers. Ten distinct missense mutations in exon 30 or 31 of the CREBBP gene had been found in these patients who had incomplete signs for RTS.

In a recent article published by Banka et al., similar results were reported that three individuals with missense mutations in exon 30 or 31 of the CREBBP gene but not have characteristic dysmorphic features of RTS [10]. When all the cases published in the literature are evaluated together with ours, it can be concluded that different variants in the CREBP gene are responsible for different clinical phenotypes. However, in order to reach a more definite conclusion on this issue, more comprehensive studies in larger patient series are required.

As a result, the clinical features of PADs and some genetic diseases, which are mostly diagnosed in childhood, may appear in adulthood, and therefore, their diagnoses and treatments may be delayed. Although the case we presented herein had strong signs of immunodeficiency, the diagnosis was delayed, and its dysmorphic features could only be revealed by reexamination according to the results of molecular analysis. Therefore, in cases with suspected immunodeficiency, more careful evaluation should be made to reduce both mortality and morbidity and the economic burden due to late diagnosis. On the other hand, it seems imperative to raise awareness of rare diseases in all segments of society.

## Data Availability

No data were used to support this study.
